# Chemical Composition of a Novel Distillate from Fermented Mixture of Nine Anti-Inflammatory Herbs and Its UVB-Protective Efficacy in Mouse Dorsal Skin via Attenuating Collagen Disruption and Inflammation

**DOI:** 10.3390/molecules26010124

**Published:** 2020-12-29

**Authors:** Young Her, Tae-Kyeong Lee, Ji Hyeon Ahn, Soon Sung Lim, Beom-Goo Kang, Jung-Seok Park, Bora Kim, Hyejin Sim, Jae-Chul Lee, Hyun Sook Kim, Tae Heung Sim, Hyun Sam Lee, Moo-Ho Won

**Affiliations:** 1 Department of Dermatology, Kangwon National University Hospital, Kangwon National University School of Medicine, Chuncheon, Gangwon 24289, Korea; youngderma@knuh.or.kr; 2 Department of Biomedical Science and Research Institute for Bioscience and Biotechnology, Hallym University, Chuncheon, Gangwon 24252, Korea; tk-lee@hallym.ac.kr; 3 Department of Neurobiology, School of Medicine, Kangwon National University, Chuncheon, Gangwon 24341, Korea; jh-ahn@ysu.ac.kr (J.H.A.); nbrkim17@gmail.com (B.K.); janny20@naver.com (H.S.); anajclee@kangwon.ac.kr (J.-C.L.); 4 Department of Physical Therapy, College of Health Science, Youngsan University, Yangsan, Gyeongnam 50510, Korea; 5 Department of Food Science and Nutrition, College of Natural Sciences, Hallym University, Chuncheon 24252, Korea; limss@hallym.ac.kr; 6 Department of Biochemistry, College of Medicine, Hallym University, Chuncheon 24252, Korea; kbgda@hallym.ac.kr; 7 Department of Physical Education, College of Natural Science, Hallym University, Chuncheon 24252, Korea; 41920@hallym.ac.kr; 8 Leefarm Co., Ltd., Hongcheon, Gangwon 25117, Korea; K18860@naver.com (H.S.K.); 119ato@naver.com (T.H.S.)

**Keywords:** distillation, fermentation, natural herb complex, skin protection, UVB

## Abstract

Since ancient times, various herbs have been used in Asia, including Korea, China, and Japan, for wound healing and antiaging of the skin. In this study, we manufactured and chemically analyzed a novel distillate obtained from a fermented mixture of nine anti-inflammatory herbs (*Angelica gigas*, *Lonicera japonica*, *Dictamnus dasycarpus Turcz.*, *D. opposita Thunb.*, *Ulmus davidiana var. japonica*, *Hordeum vulgare var. hexastichon Aschers.*, *Xanthium strumarium L*., *Cnidium officinale*, and *Houttuynia cordata Thunb.*). The fermentation of natural plants possesses beneficial effects in living systems. These activities are attributed to the chemical conversion of the parent plants to functional constituents which show more potent biological activities. In our current study, the distillate has been manufactured after fermenting the nine oriental medical plants with *Lactobacillus fermentum*, followed by distilling. We analyzed the chemical ingredients involved in the distillate and evaluated the effects of topical application of the distillate on ultraviolet B (UVB)-induced skin damage in Institute of Cancer Research (ICR) mice. Topical application of the distillate significantly ameliorated the macroscopic and microscopic morphology of the dorsal skin against photodamage induced by UVB radiation. Additionally, our current results showed that topical application of the distillate alleviated collagen disruption and reduced levels of proinflammatory cytokines (tumor necrosis factor alpha and interleukin 1 β expressions) in the dorsal skin against UVB radiation. Taken together, our current findings suggest that the distillate has a potential to be used as a material to develop a photoprotective adjuvant.

## 1. Introduction

Skin is the outermost organ of a human body, and it is the first gateway to protect against external toxic substances, infections, and ultraviolet (UV) rays [1]. Excessive UV radiation penetrates the skin and produces reactive oxygen species (ROS) in cells [2,3]. ROS lead to cellular oxidative stress and prolong inflammation in the skin [4]. Photodamaged skin is characterized by loss of tone and resilience, roughness, dryness, hyperpigmentation, erythema, and formation of deep wrinkles in the skin [5].

The mechanisms of UV-induced skin damage involve inflammation by inflammatory cytokines and collagen degradation by collagenases [6]. Among the inflammatory cytokines, proinflammatory cytokines such as interleukin (IL)-1β and tumor necrosis factor alpha (TNF-α) stimulate the generation and accumulation of ROS and, furthermore, increase the expression of matrix metalloproteinases (MMPs), which accelerate structural and functional destruction of the extracellular matrix (ECM) and lead to collagen disruption [7].

For the above mentioned reasons, many studies have focused on finding effective agents that have anti-inflammatory properties and prevent collagen breakdown in order to effectively prevent skin damage caused by UV rays. In the East, including China and Korea, various plants that have anti-inflammatory effects have been used for preventing skin photodamage and maintaining skin health since ancient times. Accumulated experimental data have demonstrated beneficial effects of various plants. For example, many plants have been proven to show anti-inflammatory effects on the skin as follows: *Angelica gigas* [8], *Lonicera japonica* [9], *Dictamnus dasycarpus Turcz.* [10], *D. opposita Thunb.* [11], *Ulmus davidiana var. japonica* [12], *Hordeum vulgare var. hexastichon Aschers.* [13], *Xanthium strumarium L*. [14], *Cnidium officinale* [15], and *Houttuynia cordata Thunb* [16]. In addition, it has been demonstrated that fermented natural products bring diverse advantageous properties, such as anti-inflammatory, antioxidant, and antiadipogenic effects [17,18]. Especially, it has been reported that essential oils distillated from natural resources exert a protective effect against UV-induced skin damage [19].

The development of products from various plants that have been traditionally used until now is in line with modern consumers’ desire for products that are eco-friendly and have no side effects. In this regard, we manufactured a novel distillate after fermentation of a mixture of nine anti-inflammatory herbs (*Angelica gigas*, *Lonicera japonica*, *Dictamnus dasycarpus Turcz.*, *D. opposita Thunb.*, *Ulmus davidiana var. japonica*, *Hordeum vulgare var. hexastichon Aschers.*, *Xanthium strumarium L*., *Cnidium officinale*, and *Houttuynia cordata Thunb.*). Furthermore, we chemically analyzed the distillate. The ultimate purpose of the present study was to examine the protective effect of the distillate against skin damage induced by ultraviolet B (UVB) and its mechanism in mice.

## 2. Results

### 2.1. Analysis of the Distillate

**Figure 1:**
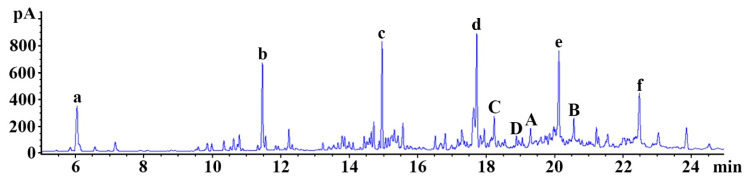
Representative gas chromatography (GC) chromatogram of the distillate. 2, 6, 10-trimethyldodecane (A; C_15_H_32_; retention time, 17.65 min; peak area, 3.69%), 2, 6, 11, 15-tetramethylhexadecane (B; C_20_H_42_; retention time, 20.58 min; peak area, 2.45%), n-heptadecane (C; C_17_H_36_; retention time, 18.25 min; peak area, 2.02%), and n-docosane (D; C_22_H_46_; rettention time, 19.31 min; peak area, 1.64%) are identified as important ingredients. a–f: siloxane compounds (impurities derived from the column).

To detect chemical components of the distillate, gas chromatography/mass spectrometry (GC/MS) was carried out (Figure 1). The chemical ingredients of the distillate are listed in Table 1. Among them, 2, 6, 10-trimethyldodecane (A; C_15_H_32_; retention time, 17.65 min; peak area, 3.69), 2, 6, 11, 15-tetramethylhexadecane (B; C_20_H_42_; retention time, 20.58 min; peak area, 2.45%), n-heptadecane (C; C_17_H_36_; retention time, 18.25 min; peak area, 2.02%), and n-docosane (D; C_22_H_46_; retention time, 19.31 min; peak area, 1.64%) were identified as important ingredients of the distillate (Figure 1 (A–D) and Table 1). Six siloxanes (Si-O-S; a functional group in organosilicon compounds) were detected, which are regarded as impurities derived from the column (Figure 1 (a–f)).

### 2.2. Skin Damage

**Figure 2:**
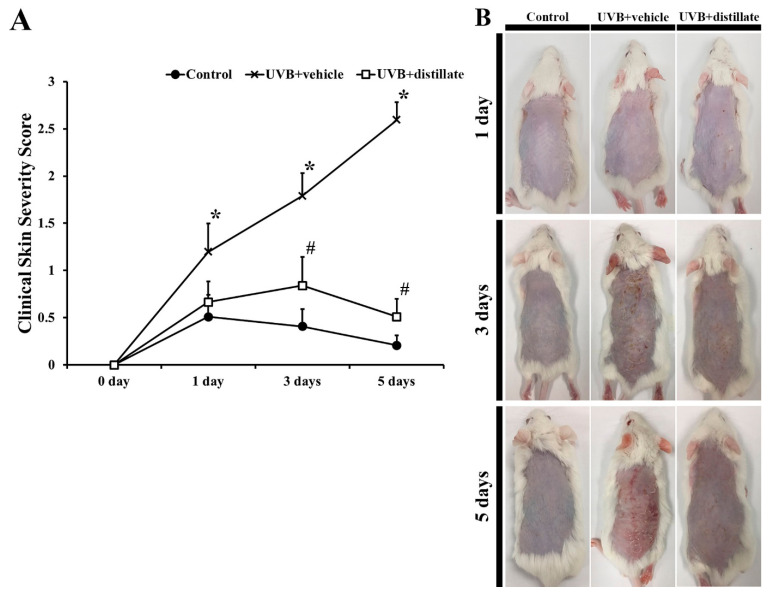
(**A**) The clinical skin severity score observed in the control, UVB + vehicle, and UVB + distillate groups at 1 day, 3 days, and 5 days after UVB exposure. The clinical skin severity score in the UVB + vehicle group was significantly increased from 1 day after UVB irradiation, however, the score in the UVB + distillate group was significantly decreased from 3 days to 5 days after UVB exposure compared to that in the UVB + vehicle group. The data represent mean ± standard errors of means (*n* = 10/group, * *p* < 0.05 vs. control group; ^#^ *p* < 0.05 vs. UVB + vehicle group). (**B**) Photos of the dorsal skin of the control, UVB + vehicle, and UVB + distillate groups at 1 day, 3 days, 5 days, and 7 days after UVB exposure.

The degree of dorsal skin damage, including scarring/dryness, edema, erythema/hemorrhage, and excoriation/erosion, was evaluated based on the clinical skin severity score (Figure 2). In the control group, the clinical skin severity score was maintained under 0.5 for 7 days (Figure 2A). However, in the UVB + vehicle group, the clinical skin severity score was significantly increased from 1 day to 5 days after UVB exposure compared with that in the control group (Figure 2A,B). In the UVB + distillate group, the skin severity score was lower at 1 day after UVB exposure and significantly decreased at 3 days and 5 days after UVB exposure compared with that in the UVB + vehicle group, although the skin severity score was higher than that in the control group (Figure 2A,B).

### 2.3. Epidermal Thickening

**Figure 3:**
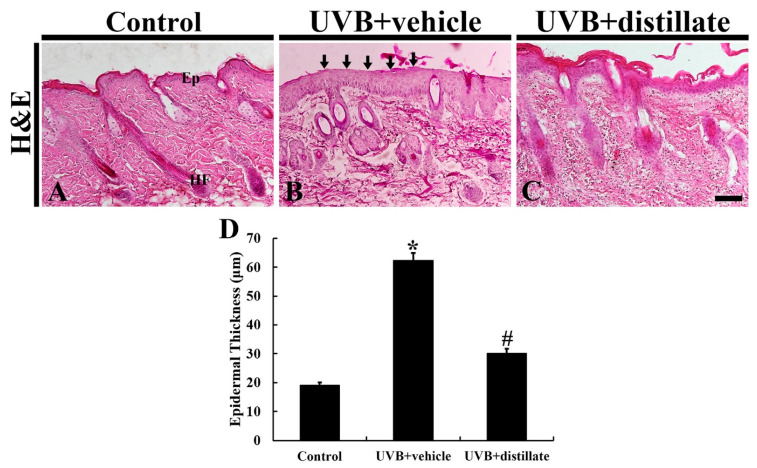
H and E staining of mouse dorsal skin in the control (**A**), UVB + vehicle (**B**), and UVB + distillate (**C**) groups at 5 days after UVB exposure. In the UVB + vehicle group, the thickness of the epidermis was significantly increased (arrows) at 5 days after UVB exposure. However, in the UVB + distillate group, the epithermal thickness was significantly reduced. Ep, epidermis; HF, hair follicle. Scale bar = 100 μm. (**D**) The mean epidermal thickness in the control, UVB + vehicle, and UVB + groups at 5 days after UVB exposure. The data represent mean ± standard errors of means (*n* = 10/group, * *p* < 0.05 vs. control group; ^#^ *p* < 0.05 vs. UVB+vehicle group).

At 5 days after UVB irradiation, epidermal thickness was measured by using hematoxylin and eosin (H and E) staining (Figure 3). In the control group, the mean thickness of the epidermis was 19.37 μm (Figure 3A,D). In the UVB-vehicle group, the hyperplasia and hypertrophy of the epidermis were shown, and the mean epidermal thickness was 63.37 μm, which was significantly increased by 327.1% compared with that in the control group (Figure 3B,D). However, in the UVB-distillate group, the mean epidermal thickness was 30.58 μm, which was significantly reduced by 207.2% compared to that in the UVB-vehicle group, showing that the epidermis showed weaker hypertrophy and hyperplasia than that in the UVB-vehicle group (Figure 3C,D).

### 2.4. Immunoreactivities of Collagen I and III

**Figure 4:**
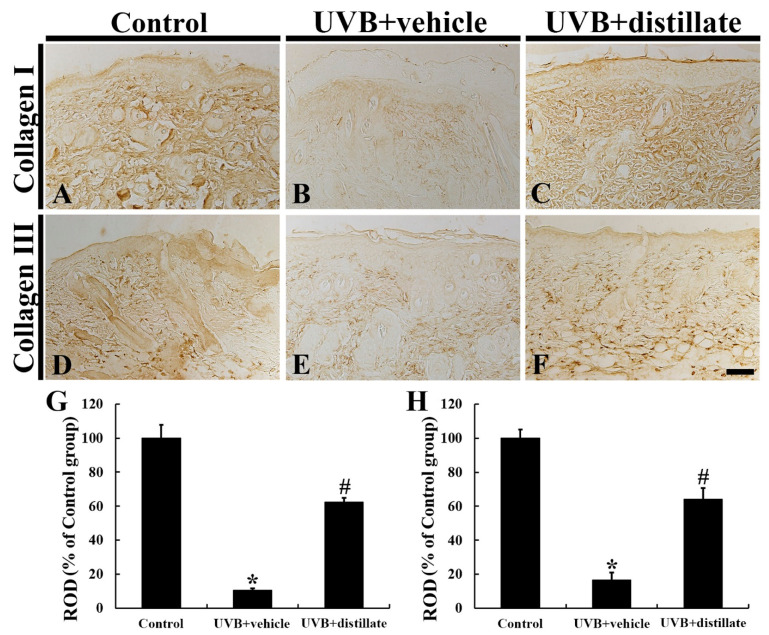
Collagen I (**A**–**C**) and III (**D**–**F**) immunohistochemistry in the dorsal skin of the control (**A**,**D**), UVB + vehicle (**B**,**E**), and UVB + distillate (**C**,**F**) groups at 5 days after UVB exposure. In the UVB + vehicle group, collagen I and III immunoreactivity in the dermis was significantly decreased compared with that in the control group. However, collagen I and III immunoreactivity in the UVB + distillate group was markedly increased compared with that in the UVB + vehicle group. Scale bar = 100 μm. (**G**,**H**) Relative optical density (ROD) of collagen I (**G**) and III (**H**) immunoreactivity in the dorsal skin as percentage of the control group. The data represent mean ± standard errors of means (*n* = 10/group, * *p* < 0.05 vs. control group; ^#^ *p* < 0.05 vs. UVB + vehicle group).

In the control group, a very dense array of collagen I immunoreactive structures was shown in the dermis of the mouse dorsal skin (Figure 4A). In the UVB + vehicle group, collagen I immunoreactive structures were disrupted and reduced in density, showing that the collagen I immunoreactivity was significantly decreased by 89.7% in the dermis when compared with that in the control group (Figure 4B,G). However, in the UVB + distillate group, collagen I immunoreactivity in the dermis was significantly improved (62.2% of the control group) compared with that in the UVB + vehicle group (Figure 4C,G).

Collagen III immunoreactive structures were densely distributed in the dermis of the mouse dorsal skin in the control group (Figure 4D). In the UVB + vehicle group, collagen III immunoreactivity was significantly decreased by 83.6% compared with that in the control group (Figure 4E,H). However, in the UVB + distillate group, collagen III immunoreactivity in the dermis was significantly increased (63.9% of the control group) compared to that in the UVB + vehicle group (Figure 4F,H).

### 2.5. Immunoreactivities of TNF-α and IL-1β 

**Figure 5:**
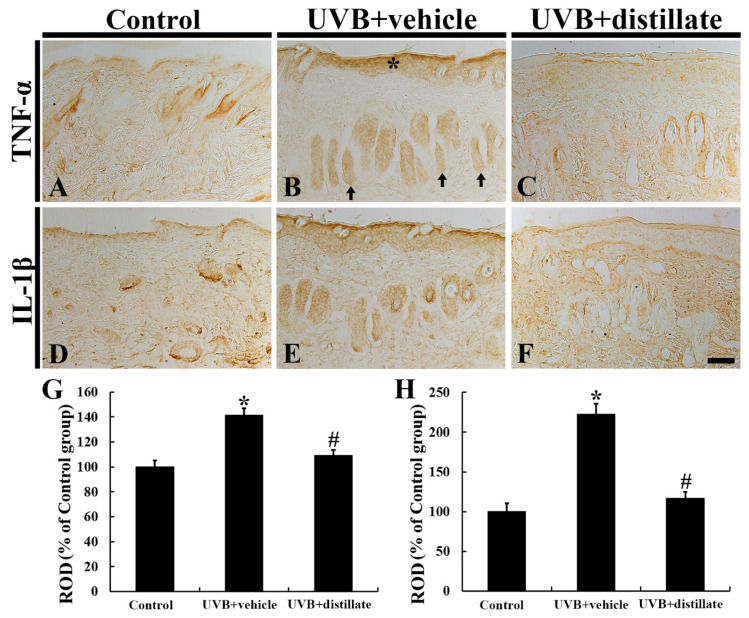
Tumor necrosis factor alpha (TNF-α) (**A**–**C**) and interleukin (IL-1β) (**D**–**F**) immunohistochemistry in mouse dorsal skin in the control (**A**,**D**), UVB + vehicle (**B**,**E**), and UVB + distillate (**C**,**F**) groups at 5 days after UVB exposure. TNF-α and IL-1β immunoreactivity in the UVB + vehicle group was significantly increased in the epidermis (asterisk) and hair follicles (arrows). However TNF-α and IL-1β immunoreactivity in the UVB + distillate group was significantly reduced compared to that in the UVB + vehicle group. Scale bar = 100 μm. (**G**,**H**) ROD of TNF-α (**G**) and IL-1β (**H**) immunoreactivity in the dorsal skin as percentage of the control group. The data represent mean ± standard errors of means (*n* = 10/group, * *p* < 0.05 vs. control group; ^#^ *p* < 0.05 vs. UVB + vehicle group).

TNF-α immunoreactivity in the control group was easily found throughout all layers in the mouse dorsal skin (Figure 5A). In the UVB + vehicle group, TNF-α immunoreactivity was significantly increased in the epidermis and hair follicles, showing that its relative optical density (ROD) was 141.7% of that in the control group (Figure 5B,G). However, TNF-α immunoreactivity in the UVB + distillate group was significantly reduced, showing that its ROD was 109.8% of that in the control group (Figure 5C,G).

IL-1β immunoreactivity in the control group was also shown in all layers (Figure 5D). In the UVB + vehicle group, IL-1β immunoreactivity was significantly enhanced in the epidermis and hair follicles, showing that its ROD was increased by 121.8% (223.8% of the control group) when compared with that in the control group (Figure 5E,H). However, in the UVB + distillate group, IL-1β immunoreactivity was significantly decreased (its ROD: 116.7% of that in the control group), but it was not significantly different from that in the control group (Figure 5F,H).

## 3. Discussion

In this study, our results showed that topical administration of the distillate attenuated UVB-induced epidermal hyperplasia and clinical changes including erythema, crust, and dryness in the mouse dorsal skin. Moreover, the present results showed that UVB-induced collagen breakdown in the dermis was ameliorated by the distillate treatment, showing that increased immunoreactivities of proinflammatory cytokines (TNF-α and IL-1β) after UVB exposure were significantly reduced by the distillate. This finding indicates that UVB-induced skin damage was significantly protected by the distillate, which may have anti-inflammatory effects that result in the protection against collagen breakdown. This suggestion is supported by a paper reporting that TNF-α augments collagenolytic activity of MMP-1, possibly through upregulation of MMP-3 leading to gradual loss of type I collagen in human skin [20].

Recently, it has been reported that products from various combinations of plants alleviate skin damage caused by UV rays [21,22]. In this sense, we have expected that a mixture acting as a photoprotector through anti-inflammatory effects results in a decrease in expression of proinflammatory cytokines including IL-1 and TNF-α in the dorsal skin of a mice model of UVB-induced skin damage. In this study, we used a mixture of nine medicinal plants known to have anti-inflammatory effects, as described in Table 2. The plants used in this study have long been used in Korea because of their excellent effects on suppressing inflammation. A recent study has demonstrated that *Angelica gigas* acts to improve inflammation by inhibiting mitogen-activated protein kinase (MAPKs) and nuclear factor-κB pathway (NF-κB) when it is applied to animal models of atopic dermatitis [8]. In addition, it has been reported that *Lonicera japonica* inhibits the NF-κB and signal transducer and activator of transcription 3 pathway (STAT3) in acute inflammation through a component called chrysoeriol [23]. Furthermore, Choi et al. (2019) have demonstrated that *Dictamnus dasycarpus Turcz* has an anti-inflammatory effect through lowering inflammatory cytokines such as interferon-γ and interleukin (IL)-17 [10]. As another example, avarol, a marine sesquiterpenoid hydroquinone derived from *Dysidea avara* (Dysideidae family), displays beneficial properties such as anti-inflammatory and antioxidant effects and it has been suggested as a material possessing potential UVB photoprotective candidates [24,25].

The distillate used in our current study was distillated from fermentation of the mixture with *Lactobacillus fermentum*. The lactic acid bacteria (genus *Lactobacillus*) belong to Gram-positive microorganisms and generate lactic acid via anaerobic respiration [26]. Among the lactic acid bacteria, *Lactobacillus fermentum* has been commonly utilized for fermentation of foods and/or beverages [27,28]. Fermented products bring beneficial effects. For example, a precedent study demonstrated that fermented *Sophora flavescens* (Fabaceae family) enhanced anti-inflammatory effects compared to nonfermented *Sophora flavescens* [17]. Furthermore, Hwang et al. (2019) showed significant improvements of antioxidant, anti-inflammatory, and antiadipogenic attributes by hydroponically cultivated Korean *Panx ginseng* (Araliaceae family) which resulted from fermentation [18]. In addition, previous studies have demonstrated that an essential oil derived from a natural resource possesses beneficial effects. For example, hydro-distillated essential oil derived from a mixture of two medical herbs belonging to the Caprifoliaceae family (*Nardostachys chinensis* and *Valeriana officinalis*) shows antimicrobial and antioxidant activities [29], and an essential oil derived from *Syzygium aromaticum* L. (Myrtaceae family) extract protects against skin damage from UVB irradiation [19].

In our current study, the chemical components of the distillate contained 2,6,10-trimethyldodecane, 2,6,11,15-tetramethylhexadecane, n-heptadecane, and n-docosane as important ingredients. Additionally, the distillate contained a minor quantity of n-nonadecane (C_19_H_40_; retention time, 19.07 min; peak area, 0.56%) and rose oxide (C_10_H_18_O; retention time, 18.48 min; peak area, 0.51%). The biological activities of these compounds have been poorly investigated, however, the leaf extract of *Hibiscus rosa sinensis* Linn (the Malvaceae family) containing the n-nonadecane was reported to display anti-inflammatory activity [30]. Besides, rose oxide contained in the distillate was demonstrated to have anti-inflammatory activity [31]. In detail, treatment with rose oxide ameliorated the volume of edema via reducing IL-1β expression in a rat model of carrageenan-induced paw edema [31]. Based on the above and current studies, some components contained in the distillate may protect against UVB-induced damage.

Ultraviolet radiation is classified into three types: (1) UVA (wave length, 320–400 nm), (2) UVB (wave length, 290–320 nm), and (3) UVC (wave length, 200–280 nm) [32]. The amount of UVB radiation reaching the surface of the earth is less than that of UVA radiation, but UVB radiation is more intense and capable of inducing acute and chronic damage to the skin by triggering DNA damage and excessively accumulating ROS [33]. ROS generated by UVB can affect mitogen-activated protein kinase signaling and activate nuclear factor-κ B and activator protein-1 to release inflammatory cytokines, including TNF-α and interleukin IL-1β [34]. In addition, an increase in MMP activity by ROS production results in the destruction of the ECM via collagen breakdown [34,35,36], and UV-exposed fibroblasts evoke the degradation of collagen type I and type III, which exist predominantly in the dermis and interfere in the production of collagens [7,37]. Thereafter, UVB irradiation leads to erythema, cutaneous edema, hyperplasia, leukocyte infiltration, dilation of blood vessels in the dermis, and their vascular hyperpermeability, resulting eventually in epidermal proliferation and skin thickening [38,39]. Generally, photo damage of the skin manifests as skin thickening, reduction in dermal elasticity, and wrinkle formation. These phenomena are fundamentally associated with change and reduction in collagen type I and III [40].

Taken together, in this study, the topical application of the distillate notably inhibited the increase of IL-1β and TNF-α in UVB-exposed mouse dorsal skin and protected against collagen breakdown from UVB exposure. Based on the present results, we suggest that the distillate may be used as a photoprotective adjuvant.

## 4. Materials and Methods

### 4.1. Preparation of the Distillate

Firstly, we prepared a mixture of nine medicinal herbs that have anti-inflammatory properties. As shown in Table 2, necessary parts (50 g per plant) were collected from the nine plants by the Leefarm Co., Ltd., (Hongcheon, Korea). Next, to obtain the fermented mixture, the mixture was added to pure water (1 L) and fermented with *Lactobacillus fermentum* at 24 °C for 20 days. Finally, the fermented mixture was centrifuged, and, to obtain the distillate, the supernatant was vacuum evaporated (temperature, 4 °C; pressure, 80 Pa) for 2 h. The feeding rate was 5 mL/min, and the rotational speed of the roller wiper was 200 rpm.

### 4.2. Analysis of the Distillate

#### 4.2.1. Chemical and Reagent

GC/MS-grade hexane and ethyl ether were purchased from Sigma Aldrich (St. Louis, MO, USA).

#### 4.2.2. Sample Preparation

We placed a 4 mL sample into a 10 mL glass vial. Subsequently, 2 mL of hexane and 2 mL of ethyl ether were added. The mixture was strongly shaken for 1 min by hand and set aside to wait 30 min for separation. A volume of 3 mL of the upper layer was transferred into a 2 mL Eppendorf vial containing 20 mg of anhydrous sodium sulfate. The vial was manually shaken for 1 min and centrifuged for 3 min at 10,000 rpm. Finally, the sample was transferred to a crimp-cap vial for injection into the gas chromatograph.

#### 4.2.3. GC

Gas chromatographic analysis was performed on a 6890N (Agilent Technologies Inc., Santa Clara, CA, USA) series gas chromatograph equipped with a splitless injector (250 °C) and FID operated at 250 °C. A DB-5ms column (length, 30 mm; diameter, 0.25 mm, 0.25 µm) was used. We used the following operating conditions: 5 min at 50 °C hold, then from 50 °C to 210 °C at 10 min (injector temperature, 250 °C; detector temperature, 280 °C; carrier gas, hydrogen; 1.0 mL/min).

#### 4.2.4. GC/MS

An Agilent 5975C (Agilent Technologies Inc., Santa Clara, CA, USA) equipped with an Agilent 7890A (Agilent Technologies Inc., Santa Clara, CA, USA) with a split/splitless injector was used for GC/MS. The samples (5 μL) were injected in split mode (ratio 50:1). A DB-5ms column (30 × 0.25 mm, 0.25 μm) was employed with helium (purity 99.999 %) as a carrier gas at constant flow rate (1.0 mL/min). The temperature of the column was programmed as the GC method. The temperatures of the ionization source and transfer line were 280 °C. The electron energy was 70 eV. Mass spectra were obtained by automatic scanning of the mass range m/s 50–500 atomic mass units.

### 4.3. Experimental Animals

A total number of 30 ICR mice (body weight, 30; 35 ± 5 g) at 8–9 weeks old were provided by Central Lab. Animal Inc. (Seoul, Republic of Korea). They were housed under conventional housing conditions of which the ambient temperature was 23 ± 3 °C and relative humidity was 55 ± 5% under a 12 h light/dark cycle. Free access to food and water was allowed. All experimental protocols including animal handling and use were reviewed and approved (approval no. KW-200121-2) by Institutional Animal Care and Use Committee located in Kangwon National University (Chuncheon, Korea). All procedures for animal handling and care were done in compliance with the “Current International Laws and Policies” from NIH Guide for the Care and Use of Laboratory Animals (NIH Publication No. 85–23, 1985, revised 1996). All experiments were carried out minimizing the numbers of animals used and their suffering by the procedures used in the present study.

### 4.4. Experimental Groups, Treatment of Distillate, and UVB Irradiation

ICR mice were randomly divided into 3 groups (*n* = 10/group) as follows: (1) control group, (2) UVB-exposed and vehicle-treated group (UVB + vehicle group), and (3) UVB-exposed and distillate-treated group (UVB + distillate). Dorsal hairs of mice were shaved at 3 days before UVB exposure. Mice in the UVB and UVB + distillate groups received 200 μL of topical applications of distilled water (DW; vehicle) or distillate to dorsal skin twice a day for 3 days before the start of UVB exposure, and before and after the UVB exposure for the remaining 5 days. The source of UVB was a UVM-225D Mineralight UV Display Lamp (UVP, Phoenix, AZ, USA), and UVB irradiation exposure was applied at 150 mJ/cm^2^ for 3 min for 5 days.

### 4.5. Assessment of Clinical Severity of Skin Injury 

The clinical severity of the dorsal skin lesion (dermatitis) was scored by diagnostic criteria at microscopic level, which is normally assessed in human dermatitis [41]. In our current study, the severity of dermatitis was assessed 4 times a week by 2 independent dermatologists. Change in skin conditions including erythema/hemorrhage, edema, scarring/dryness, and excoriation/erosion was estimated as 0 (none), 1 (mild), 2 (moderate), and 3 (severe). In this study, individual score was taken as the dermatitis score.

### 4.6. Tissue Processing for Histology

To obtain the dorsal skin, the mice (*n* = 10/group) were anesthetized by intraperitoneal injection of pentobarbital sodium (40 mg/kg) (JW Pharm. Co., Ltd., Korea) at 5 days after the treatments. The anesthetized mice were transcardially perfused with 0.1 M phosphate-buffered saline (PBS) (pH 7.4) and fixed with 4% paraformaldehyde solution (in 0.1 M PB, pH 7.4). The dorsal skin tissues of the mice were removed, and the tissues were further fixed in the same fixative for one day and embedded in paraffin. Finally, the embedded tissues were sectioned into 8 µm thickness in a microtome (Leica, Wetzlar, Germany).

### 4.7. Hematoxylin and Eosin Staining 

Histopathological changes of the dorsal skin were evaluated by H and E staining. In short, the sections were stained with hematoxylin and eosin (Sigma-Aldrich, St. Louis, MO, USA), dehydrated with serial ethanol. Finally, the stained sections were mounted with Canada balsam from Kanto Chemical (Tokyo, Japan) [42].

### 4.8. Immunohistochemistry 

The dorsal skin tissue sections were immunohistochemically stained according to our published method [43]. In short, the sections were treated with 0.3% H_2_O_2_ (in 0.1 M PBS, pH 7.4) and immersed in 10% normal goat, horse, or rabbit serum (Vector Laboratories, Inc., Burlingame, CA, USA) (in 0.05 M PBS) for 0.5 h at room temperature. Next, the sectioned dorsal skin tissues were reacted in solution of primary antibodies (rabbit anti-type I collagen (diluted 1:400) (Abcam, Cambridge, MA, USA), mouse anti-type III collagen (diluted 1:300) (Abcam, Cambridge, MA, USA), rabbit anti-interleukin (IL)-1β (diluted 1:200) (Santa Cruz Biotechnology, Santa Cruz, CA, USA), and rabbit anti-tumor necrosis factor (TNF)α (diluted 1:1000) (Abcam, Cambridge, MA, USA)) at 4 °C for 24 h. Thereafter, these sections were subjected to biotinylated horse anti-mouse IgG (diluted 1:250, Vector, CA, USA), goat anti-rabbit IgG (diluted 1:250, Vector, CA, USA), or rabbit anti-goat IgG (diluted 1:250, Vector, CA, USA) as secondary antibody. Lastly, these sections were reacted in solution of avidin-biotin complex (diluted 1:300, Vector, CA, USA). Finally, these sections were visualized by reacting them with solution of 3, 3′-diaminobenzidine tetrahydrochloride (DAB) (Sigma, MO, USA) (in 0.1 M PBS, pH 7.4). Each negative control test was done to establish the specificity of each immunostaining with pre-immune serum instead of each primary antibody. We found that each test showed no immunoreactivity in the observed sections (data not shown).

### 4.9. Data Analyses

Immunoreactivity of collagen I, collagen III, IL-1β, and TNFα was quantitatively analyzed according to our published protocol [43]. Briefly, we captured a digital image of each immunoreactive structure in the dorsal skin by using a light microscope (BX53, Olympus, Tokyo, Japan). Each immunoreactivity was evaluated as relative immunoreactivity (RI) as follows. Firstly, based on an optical density (OD) of the image, OD was obtained after transforming the color image into mean gray level by using a formula: OD = log (256/mean gray level). Next, the background was removed from the areas around the measured areas. Finally, RI of collagen I, collagen III, IL-1β, and TNFα in the dorsal skin was calibrated as % after background was subtracted and compared with the control group designated as 100% by Adobe Photoshop of version 8.0 from San Jose (CA, USA) and NIH Image software of version 1.59 from NIH (Bethesda, MD, USA).

### 4.10. Statistical Analysis

The data shown in this study represent the means ± standard error of the mean (SEM). Differences of the means among the groups were statistically analyzed by two-way analysis of variance (ANOVA) with a post hoc Bonferroni’s multiple comparison test to determine differences among groups. Statistical significance was considered at *p* < 0.05.

## Figures and Tables

**Table 1:** Compounds qualified in the distillate using gas chromatography/mass spectrometry.

Retention Time	Compound	Molecular Formula	Molecular Weight	Peak Area (%)
5.86	2,4-Dimethylhexane	C_8_H_18_	109.4	0.33
6.06	Siloxane	-	-	3.88
6.58	2,4-Dimethylheptane	C_9_H_20_	128.25	0.35
7.17	Unknown	-	-	0.67
9.86	Unknown	-	-	0.52
9.99	Unknown	-	-	0.35
10.35	Unknown	-	-	0.59
10.63	3,6-Dimethylundecane	C_13_H_28_	184.36	0.68
10.74	Octyl chloroacetate	C_10_H_19_C_l_O_2_	206.71	0.33
10.80	3,8-Dimethyldecane	C_12_H_26_	170.33	0.77
11.47	Siloxane	-	-	4.44
11.57	n-Octane	C_8_H_18_	114.23	0.66
12.25	4-Methyl-2-Undecene	C_12_H_24_	168.32	1.13
12.34	4-Methylcyclohexanone	C_7_H_12_O	112.17	0.37
13.24	n-Decane	C_10_H_22_	142.28	0.34
13.41	2-Methyl-1,5-hexadiene	C_7_H_12_O	96.17	0.32
13.55	n-Undecane	C_11_H_24_	156.31	0.52
13.68	3,7-Dimethyldecane	C_12_H_24_	170.33	0.42
13.80	5-Butylnonane	C_13_H_28_	184.36	0.98
13.88	3-Methyl-2-butene-1-ol (Prenol)	C_5_H_10_O	86.132	0.64
14.01	Unknown	-	-	0.50
14.45	Terpinen-4-ol	C_10_H_18_O	154.25	0.68
14.53	4,6-Dimethylundecane	C_13_H_28_	184.36	0.46
14.60	Unknown	-	-	0.71
14.66	Unknown	-	-	0.78
14.73	Unknown	-	-	1.33
14.89	3,5-Dimethyloctane	C_10_H_22_	142.28	0.42
14.97	Siloxane			4.56
15.07	3,9-Dimethylundecane	C_13_H_28_	184.36	0.61
15.15	5,7-Dimethylundecane	C_13_H_28_	184.36	0.66
15.23	1,4-Dimethylcyclooctane	C_7_H_12_O	140.27	0.53
15.26	2,3-Dimethyl-3-heptene	C_9_H_18_	126.24	0.87
15.33	Unknown	-	-	1.50
15.43	Hexyl octyl ether	C_14_H_30_O	214.39	0.83
15.58	n-Tridecane	C_13_H_28_	184.36	1.45
16.52	n-Tetradecane	C_14_H_30_	198.39	0.64
16.68	n-Pentadecane	C_15_H_32_	212.42	0.66
16.82	4-Methyldodecane	C_13_H_28_	184.36	0.87
17.18	Unknown	-	-	0.66
17.30	Unknown	-	-	1.39
17.38	Unknown	-	-	0.44
17.45	n-Hexadecane	C_16_H_34_	226.41	0.43
17.65	2,6,10-Trimethyldodecane	C_15_H_32_	212.41	3.69
17.74	Siloxane	-	-	5.98
17.84	4,6-Dimethyldodecane	C_14_H_30_	198.39	0.89
17.96	2,3,5,8-Tetramethyldecane	C_14_H_30_	198.39	1.05
18.17	Unknown	-	-	0.58
18.25	n-Heptadecane	C_17_H_36_	240.48	2.02
18.36	1-Butyl-2-propylcyclopentane	C_13_H_26_	182.35	0.67
18.48	Rose oxide	C_10_H_18_O	154.25	0.51
18.56	4-Methyl-1-decene	C_11_H_22_	154.29	0.74
18.89	(2-Methylbutyl)cyclopentane	C_10_H_20_	140.27	0.66
18.96	2,6,10-Trimethyldodecane	C_15_H_32_	212.41	0.74
19.07	n-Nonadecane	C_19_H_40_	268.52	0.55
19.31	n-Docosane	C_22_H_46_	310.6	1.64
19.44	2-Ethyl-3-(isobutyryloxy)hexyl-2-methylpropanoate	C_16_H_30_O_4_	286.41	0.75
19.48	Unknown	-	-	0.41
19.61	Unknown	-	-	1.27
19.74	Unknown	-	-	0.76
19.79	n-Pentacosane	C_25_H_52_	252.69	0.64
19.86	Unknown	-	-	1.00
19.92	Unknown	-	-	0.53
19.99	1-Isobutyl-4-isopropyl-3-isopropyl-2,2-dimethylsuccinate	C_16_H_30_O_4_	286.41	1.37
20.02	2-Octadecyloxyethanol	C_20_H_42_O_2_	314.5	0.82
20.14	Siloxane	-	-	6.26
20.21	n-Hexacosane	C_26_H_54_	366.71	0.78
20.27	2-Methylhexadecane	C_17_H_36_	240.47	0.59
20.36	n-Pentatriacontane	C_35_H_72_	492.96	0.59
20.44	2,6,10,14-Tetramethylhexadecane(Phytan)	C_20_H_42_	282.5	0.54
20.48	n-Tricosane	C_23_H_48_	324.63	0.36
20.58	2,6,11,15-Tetramethylhexadecane	C_20_H_42_	282.5	2.45
20.67	6-Butyl-1,4-cycloheptadiene	C_11_H_18_	150.26	0.33
20.72	5-Undecen-3-yne,	C_11_H_18_	150.26	0.71
20.81	3,7,11,15-Tetramethylhexadecan-1-ol (Dihydrophytol)	C_20_H_42_O_2_	298.5	0.55
20.95	2-Undecene	C_11_H_22_	154.29	0.43
21.02	11-Decyldocosane	C_32_H_66_	450.9	0.35
21.07	Unknown	-	-	0.33
21.23	Unknown	-	-	1.02
21.29	2-Hexyl-1-decanol	C_16_H_34_O	242.44	0.62
21.42	Unknown	-	-	0.09
21.51	Tetrahydrolavandulol	C_10_H_22_O	158.28	0.43
21.57	Unknown	-	-	0.87
21.72	Unknown	-	-	0.38
21.94	Unknown	-	-	0.31
22.01	Unknown	-	-	0.57
22.05	Unknown	-	-	0.58
22.14	Unknown	-	-	0.56
22.19	Unknown	-	-	0.57
22.26	Unknown	-	-	0.33
22.33	Unknown	-	-	0.61
22.38	Unknown	-	-	0.78
22.49	Siloxane	-	-	4.31
22.63	2-Octadecyloxyethanol	C_20_H_42_O_2_	314.5	0.36
23.04	1,2,4-Trimethylcyclohexane	C_9_H_18_	126.24	1.22
23.86	n-Hexatiacontane	C_36_H_74_	506.97	1.46
24.53	trans-5-Undecene	C_11_H_22_	154.29	0.70
25.25	5-Ethyl-2-methyloctane	C_11_H_24_	156.31	1.07
25.43	1-Butyl-2-propylcyclopentane	C_13_H_26_	182.35	1.19
25.82	9-Eicosene	C_20_H_40_	280.53	1.17
			Total	100.00

**Table 2:** Nine oriental medical plants used in this study.

Common Name	Botanical Name	Family	Part
Korean angelica	*Angelica gigas*	Apiaceae	Root
Japanese honeysuckle	*Lonicera japonica*	Caprifoliaceae	Bloom
Fraxinella	*Dictamnus dasycarpus Turcz.*	Rutaceae	Root
Chinese yam	*D. opposita Thunb.*	Dioscoreaceae	Root
Japanese elm	*Ulmus davidiana var. japonica*	Ulmaceae	Bark
Malt	*Hordeum vulgare var. hexastichon Aschers.*	Gramineae	Seed
Rough cocklebur	*Xanthium strumarium* L.	Asteraceae	Seed
Chunkung	*Cnidium officinale*	Apiaceae	Root
Fish mint	*Houttuynia cordata Thunb*.	Saururaceae	Leaf

## Data Availability

The data presented in this study are available on request from the corresponding author.
